# Strain perceptibility of elements on the diffusion in Zr-based amorphous alloys

**DOI:** 10.1038/s41598-020-61023-0

**Published:** 2020-03-12

**Authors:** A. Y. Lee, S. Y. Kim, H. Jang, Y. D. Kim, F. Spieckermann, G. Wilde, J. Eckert, M. H. Lee

**Affiliations:** 1 Advanced Process and Materials R&D Group, Korea Institute of Industrial Technology, Incheon, 21999 Korea; 2 Department of Materials Science and Engineering, Hanyang University, Seoul, 04763 Korea; 3 Department of Materials Science, Chair of Materials Physics, Montanuniversität Leoben, A-8700 Leoben, Austria; 4 Graduate School of Molecules and Interfaces, University of Münster, 48149 Münster, Germany; 5 Erich Schmid Institute of Materials Science, Austrian Academy of Sciences, A-8700 Leoben, Austria

**Keywords:** Phase transitions and critical phenomena, Glasses

## Abstract

With the discovery of bulk metallic glasses (BMGs), there has been considerable interest in understanding their mechanical behavior. In spite of these previous observations on the relation between plastic deformation of metallic glasses and their diffusion behavior, a detailed understanding on the diffusion of BMGs is still unexplored. We evaluated the contribution of deformation-induced structural transformations (elastic, anelastic, viscoplastic or viscoelastic responsive and plastic strain) on the diffusion of Zr-based bulk metallic glasses in as-cast, elastostatically stressed and plastically deformed states. Experimental investigations of the diffusion process and the elemental distributions in the glassy alloy were performed following plastic deformation by multiple cold rolling and elastostatic cyclic compression, respectively. We compared the vacancy model and the transition state model to verify the diffusion mechanism in the deformed bulk metallic glass. The diffusion of tracer atoms, i.e., Fe, in the bulk metallic glass is affected by viscoelastic responsive strain governing the transition-state model. In contrast, the diffusion of constituent atoms, i.e., Ti, Zr, in the bulk metallic glass is dominantly affected by plastic strain governing the vacancy model. The results reveal that the diffusion behavior of bulk glassy alloys can be changed by variation of the constituent elements and applying different strain modes upon deformation.

## Introduction

The elastic and plastic responses of metallic glasses are fundamentally different from those of crystalline materials. With the discovery of bulk metallic glasses (BMGs), there has been considerable interest in understanding their mechanical behavior^1–7^. It has been shown that the viscoplastic or viscoelastic response of metallic glasses consists of two distinct parts: the anelastic part, characterized by time-delayed reversibility (recoverable) on unloading, and the viscoplastic or viscoelastic responsive part, which is irreversible (non-recoverable)^2–4^. The effect of prestraining or residual stresses on the physical properties, especially mechanical properties or plasticity, of bulk metallic glasses (BMGs) has been extensively studied^5–7^. Typically, prestraining of BMGs or BMG matrix composites can improve the plasticity of monolithic BMGs^5–7^. The residual stress distribution throughout the thickness of as-cast BMG plates is roughly parabolic with surface compression balanced by internal tension^6^.

Amorphous solids ‒ especially bulk metallic glasses (BMGs) ‒ are highly attractive for the experimental observation of the effect of strain on diffusion without macroscopic microstructural changes of the material due to the absence of clearly defined atomic planes or crystal structures^8,9^. These characteristics of amorphous alloys are closely related to their unique atomic structure, i.e., dense random packing of the constituent elements and the absence of crystalline periodicity or microstructure^8–10^, even though recent results indicate deformation-induced modifications of the medium-range order^11^.

Atomic transport and diffusion in amorphous alloys or BMGs are of considerable interest not only from a basic scientific point of view but also for various technical applications of metallic glasses^8–16^. Many reports can be found in the literature that attempt to understand the diffusion process of amorphous systems^8–16^. Moreover, the effect of strain, induced by (severe) plastic deformation (i.e., cold-rolling) on the diffusion behavior of as-cast BMGs has been investigated^13,14^. The available experimental and computational results suggest that the diffusion along shear bands during deformation may be related to geometrical constraints resulting from multi-axial stress conditions or strain-induced modifications of the MRO structure. Furthermore, a strain-induced alteration of diffusion has been experimentally observed in cold-rolled Zr-based BMG samples and numerically postulated for simple amorphous systems^14^. In spite of these preliminary observations on the relation between plastic deformation of metallic glasses and their diffusion behavior, a detailed understanding of the contribution of deformation-induced structural transformations (elastic, anelastic, viscoelastic, viscoplastic and plastic strain) on the diffusion of BMGs is still unexplored.

In the present study, we compare the diffusion behavior under different deformation modes ranging from plastically deformed specimens subjected to multiple cold-rolling cycles to samples that were elastically loaded by cyclic elastostatic compression to evaluate the change of the kinetics of diffusion that occurs under these different strain states. In particular, we investigate the effect of the stored energy for as-cast, viscoelastostatically strained and plastically deformed samples, respectively, on the diffusion behavior of Zr-based BMGs.

## Results and Discussion

### Change of structural state by different strain modes

**Figure 1:**
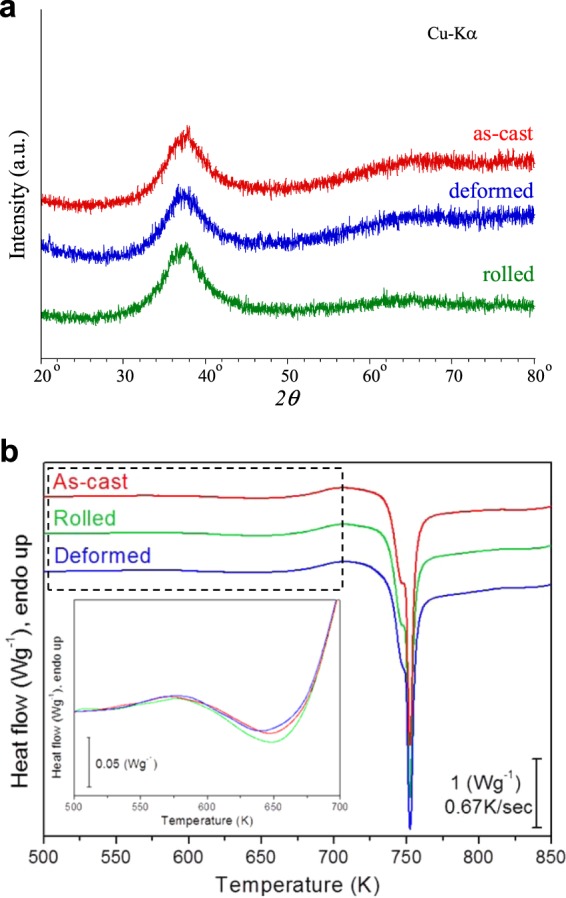
(**a**) X-ray diffraction patterns obtained for as-cast Zr_55_Ti_5_Al_10_Cu_20_Ni_10_ BMG, elastostatically loaded samples and cold-rolled, diffusion annealed specimens. (**b**) The thermal properties of the samples were measured with a differential scanning calorimeter (DSC) using constant-rate heating at 40 K/min (0.67 K/s). DSC traces obtained for as-cast Zr_55_Ti_5_Al_10_Cu_20_Ni_10_ BMG, elastostatically loaded samples and cold-rolled, diffusion annealed specimens (the scale bar corresponds to 1 W/g). (Inset) Enlarged view of the rectangular regions near to the glass transition temperature (*T*_g_) showing the exothermic heat released as a result of the reduction of disorder associated with structural relaxation (annihilation of excess free volume) during heating.

The X-ray diffraction (XRD) patterns of the as-cast, plastically cold-rolled and elastically loaded Zr_55_Ti_5_Al_10_Cu_20_Ni_10_ BMGs in Fig. 1(a) show the typical broad diffraction maxima characteristic for amorphous materials and no distinct crystalline peaks are detected within the sensitivity limits of XRD. This result corroborates that the as-cast bulk glassy alloy does not devitrify during plastic deformation by multiple cold-rolling steps. In case of elastic loading conditions realized by uniaxial compression at room temperature, macroscopic strain can be imparted without any evidence of slip steps at the surface, which are characteristic of major shear bands. To explore the possibility of room temperature elastic loading via cyclic compression, rectangular-shaped Zr-based bulk metallic glass specimens [4 mm in width and 2 mm in height (aspect ratio = 0.5)] were prepared from the as-cast ingot. The ends of the samples were carefully polished flat and normal to the longitudinal axis to assure uniform compression in the elastic range.

The thermal characteristics of the as-cast, cold-rolled, and elastically loaded (elastostatic compression) Zr_55_Ti_5_Al_10_Cu_20_Ni_10_ BMGs are shown in Fig. 1(b). The DSC traces of the as-cast, cold-rolled, and elastically loaded BMGs exhibit no significant difference despite a slight decrease of the crystallization temperature (*T*_x-as-cast_ = 734.71 ± 0.2 K, T_x-rolled_ = 734.34 ± 0.2 K, *T*_x-deformed_ = 735.80 ± 0.2 K) and the glass-transition temperature (*T*_g-as-cast_ = 671.66 ± 0.2 K, *T*_g-rolled_ = 671.87 ± 0.2 K, *T*_g-deformed_ = 673.19 ± 0.2 K) of the different specimens, respectively. The glass transition temperature, corresponding to the transition from the frozen-in to the isoconfigurational thermally relaxed structure^17^, of the elastically loaded (deformed) Zr-based BMG sample lies within the error range of the measurements during the endothermic event characterizing *T*_g_. This is similar to the previously observed small decrease of the crystallization temperature with increasing strain of plastically deformed Zr-based BMG^18,19^. However, a small increase in the enthalpy of crystallization of the cold-rolled specimen was observed (*ΔH*_x-as-cast_ = 35.19 ± 0.1 J/g, *ΔH*_x-rolled_ = 36.10 ± 0.1 J/g, and *ΔH*_x-deformed_ = 35.58 ± 0.1 J/g), which can be ascribed to the increased amount of exothermic heat due to stored energy from both plastic deformation and residual strain, as it has been demonstrated for a Cu-Zr glassy alloy^4,20^. This was explained by the fact that part of the work done during plastic deformation remains stored in the amorphous structure as strain-induced local dilatation or shear bands (increase in certain interatomic distances or so-called free volume). This has also been corroborated by measurements of the boson peak showing an enhanced magnitude after straining, indicating enhanced structural disorder and a larger amount of elastically “soft” zones with enhanced free volume^21,22^. It is supposed that no crystallization occurred upon deformation since the enthalpy of crystallization did not decrease for the cold-rolled specimens^19,23^. In addition, there is a slight change in the exothermic heat for metallic glasses after elastostatic compression which depends on the applied stress level compared to the yield stress^5,7^.

It was considered that room temperature elastostatic loading of metallic glasses at stresses of 90% of the macroscopic yield stress raises their enthalpy, but below 90% of the macroscopic yield stress, it decreases their enthalpy^5,20,24^. An enthalpy increase of less than 10% was reported for heavily plastically deformed metallic glasses^5^. In the current study, as shown in Fig. 1(b), the enthalpy of the cold-rolled samples increases by 2.58% compared to that of the as-cast material, and the enthalpy obtained from elastically loaded samples is about 1.1% larger than that of as-cast samples due to the applied loading stress of 85% of the yield stress. The enthalpy increase is proportional to the amount of loosely packed structurally disordered motifs created by the applied strain. The inset in Fig. 1(b) displays a magnified view of the DSC trace below the glass transition temperature (*T*_g_) showing the amount of exothermic heat associated with the disorder or free volume that was induced by the viscoplastic strain during elastostatic compression^5,7^. The changes in the exothermic heat flow are rather small for the elastostatically deformed samples, while a considerable change is observed for the cold-rolled specimens. The stored energy of cold work in MGs was first studied by Chen^25^, who rolled pre-relaxed Pd-based metallic glass ribbons to a reduction in thickness of 34%. Heavily cold-rolled metallic glasses, with a reduction in thickness of 50~60%, show an increase in enthalpy by 3~5%^5,25^. The application of stress below the yield strength of Cu-based and Zr-based BMGs induces structural rearrangements along with the change of the exothermic heat flow signal, as it was shown by Park *et al*. and Zhang *et al*., respectively^7,20^.

To apply elastic loading, uniaxial room temperature compression of BMGs at a constant load within the elastic regime has been carried out for several hours (~20 hr). After elastic loading, the samples show an increase in enthalpy which is associated with the viscoplastic strain component^5^. It has been reported that elastic loading can give stored energies nearly as high as those after heavy cold rolling^5,7,20^.

### Prestraining by different deformation modes

The viscoelasticity of MGs is caused by recoverable atomic adjustments occurring in response to an applied stress^24,26^. Hufnagel and Ott measured the strain in Zr_55_Ti_5_Cu_20_Ni_8_Al_10_ bulk metallic glass under uniaxial compression in the elastic regime for up to 60% of the yield strength by *in-situ* X-ray scattering^27^. They observed that elastic deformation of a metallic glass has a significant anelastic component due to atomic rearrangements^27^. It has also been shown that elastostatic compression promotes structural changes of amorphous alloys^5,7,20^. Elastostatic compression was achieved by loading-unloading cycles at room temperature, in which the samples were loaded to 85% of the yield stress, held at that stress and then unloaded^7^. The total strain caused by the elastostatic compression consists of three components corresponding to elastic strain (*ε*_*E*_), anelastic strain (*ε*_*A*_) and viscoplastic or viscoelastic responsive strain (*ε*_*V*_). Among these strain components, the elastic and anelastic strain components are fully recoverable on the removal of the applied load. In contrast, the viscoplastic or viscoelastic responsive strain component is not recoverable even after the load is removed and thus produces a permanent deformation characterized by a strain rate ($\dot{\gamma }$)^7^. The magnitudes of each strain component and their recoverability differ, depending on the composition of the samples and the relation to their structure^7^.

**Figure 2:**
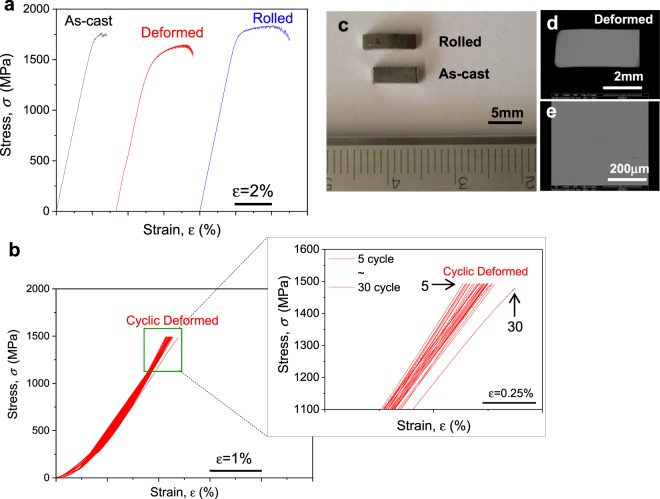
(**a**) Engineering stress-strain curves obtained from uniaxial compression tests for the as-cast Zr_55_Ti_5_Al_10_Cu_20_Ni_10_ BMG, the elastostatically deformed BMG with 2.5% thickness reduction after cyclic uniaxial compression with 30 cycles up to 85% of the yield strength at 1 × 10^–5^ s^−1^ strain rate and for the cold-rolled BMG with 15% thickness reduction after cold rolling (initial strain rate: 3×10^–4^ s^−1^). (**b**) Magnified view of the cyclic compression during elastostatic loading; the end points during each loading and unloading cycle show the effect of the loading history (pre-straining) on the strain up to 2.5%; the last cycle deforms significantly by viscoplastic or viscoelastic responsive straining within the elastic regime of the BMG. (**c**) Optical image of the cold-rolled BMG showing an overall view of the slab shape with 1.7 mm thickness (15% reduction), 2.30 mm width and 8.25 mm length that was produced by multiple cold rolling of the rectangular-shaped as-cast BMG with 2 mm thickness and width and 7 mm length. (**d**) SEM image of the elastostatically deformed BMG showing an overall cross-sectional view and a perpendicular view (**e**) of the slab shape with 2.01 mm thickness (2.5% reduction), 2.52 mm width and 4.57 mm length that was produced by cyclic compression of the rectangular-shaped as-cast BMG with 2.06 mm thickness.

It is know that the structural state of amorphous alloys determines their mechanical properties^28^. Therefore, changes in the structure due to elastostatic compression are expected to alter the properties of amorphous alloys^8^. According to our previous study, the effective strain significantly contributes to the change of the diffusivity of Fe tracer atoms in cold rolled Zr-based BMG^14^. Moreover, the contribution of the effective strain introduced by deformation is larger for ductile Zr-based BMGs than for brittle Hf-based BMGs^29^. Figure 2(a) presents a room temperature engineering stress‒strain plot of the as-cast material together with the results obtained for the pre-strained BMGs subjected to multiple cold rolling steps (plastic strain) and cyclic elastostatic compression (viscoplastic strain) at room temperature, respectively. For cyclic compression, the stress level was gradually increased within 5 cycles up to 85% of the yield stress of the as-cast sample to prevent the formation of micro-cracks or defects. The uniaxial compression test results show that the as-cast Zr-based BMG exhibits rather high strength (1769 MPa) but fails at ≤ 1.0% plastic strain. In contrast, the prestrained specimens obtained by cyclic compression show an enhancement of the compressive plasticity (plastic strain: 2.9%) with decrease the compressive strength (1646 MPa). Moreover, the prestrained specimens obtained by multiple cold rolling steps show an enhancement of the compressive plasticity (plastic strain: 3.2%) as well as the compressive strength (1837 MPa), which increase linearly with the degree of prestraining by cold rolling, as shown by the optical image in Fig. 2(c). The mechanical properties for the as-cast, multiple cold-rolled and cyclically elastostatically compressed Zr-based BMGs are summarized in Table 1. An improvement of the compressive plasticity of Zr-based monolithic BMG up to 15% strain and a strength increase up to 2.6 GPa was observed for 30% cold-rolled Zr-based BMG^23^. A similar improvement of compressive plasticity and strength due to the existence of compressive residual stresses on the surface of a shot-peened Zr_41.25_Ti_13.75_Cu_12.5_Ni_10_Be_22.5_ BMG was also reported^19,28^. Therefore, a change in the structural configurations and/or short-range order and/or different amounts of free volume due to the presence of deformation-induced residual stresses in the glass is expected.

**Table 1:** Characteristic mechanical properties: strain, strength and Young’s modulus obtained from the compression tests of as-cast, deformed (viscoelastostatically loaded) and cold-rolled (plastically deformed) Zr_55_Ti_5_Al_10_Cu_20_Ni_10_ BMGs, respectively, as also shown in Fig. 2.

Samples	Amount of deformation (%)	Yield strength (MPa)	Ultimate strength (MPa)	Yield strain (%)	Plastic strain (%)	Young’s modulus (GPa)
As-cast	0	1521 ± 0.5	1769 ± 0.5	1.81 ± 0.1	1.00 ± 0.1	85 ± 1
Deformed	2.5 ± 0.1	1168 ± 0.5	1646 ± 0.5	1.45 ± 0.1	2.85 ± 0.1	83 ± 1
Deformed*	2.5 ± 0.1	1500 ± 0.5*	—	1.6 ± 0.1*	0.25 ± 0.1**	85 ± 1
Rolled	15 ± 0.1	1577 ± 0.5	1837 ± 0.5	1.83 ± 0.1	3.17 ± 0.1	88 ± 1

*interrupted cyclic deformation, **viscoplastic or viscoelastic responsive strain.

Interestingly, as shown in Fig. 2(b), the prestrained Zr-based BMG samples subjected to cyclic elastostatic compression exhibit a deformation of 0.25% that only originates from viscoplastic or viscoelastic responsive strain. Further observations, shown in the SEM images in Fig. 2(d,e), on the lateral and normal surfaces of the cyclically elastostatically compressed prestrained samples did not show any evidence for the formation of shear bands. Moreover, the stress-strain curve of the last 30 cycles plotted in Fig. 2(b) is shifted from the other curves, indicating that the internally stored energy caused by elastostatic loading is significant in the sample suggesting the presence of viscoplastic or viscoelastic responsive strain which shows a hysteresis effect upon cycling deformation. It is well-known that when a periodic stress is applied, the structural units in the glass can be reoriented along the stress field and a relaxation phenomenon occurs at an appropriate frequency^23,24^. The increase of internal friction/higher stress indicates that a periodic external stress or strain is stochastically resonant with the atomic motions associated with the reconfiguration of clusters^30^. In such situation, atoms will move into more stable sites due to the periodic external stress^30,31^. Hysteresis is a typical characteristic feature of viscoplastic or viscoelastic responsive behavior rather than purely elastic behavior^7,24,32^ and viscoelasticity depends on the degree of structural reconfiguration^33^. Ye and co-workers have shown the existence of inelastically deformed atomic cluster zones which lead to mechanical hysteresis within the elastic region under quasi-static loading conditions^34^. Harmon *et al*. proposed reversible and irreversible configurational properties across the energy landscape associated with the transition from anelasticity to plasticity in a deformed metallic glass^35^. Hence, it is well-accepted that viscoplasticity depend on the degree of structural reconfiguration^5,7^ and is a result of the inelastic response of atoms or clusters formed in the glassy phase even in the elastic regime below the yield strength^27,34^. Therefore, the permanent deformation induced by elastostatic compression at room temperature can be regarded as “homogeneous” in agreement with previously proposed results^7,27,34^.

### Effect of different strain modes on the diffusion characteristics

The structural changes induced by the applied strain may cause a change of the diffusion behavior of BMGs. To check this, we performed diffusion experiments for the plastically deformed BMGs subjected to multiple cold rolling (prestrained by plastic strain) and for the elastically deformed BMGs obtained by cyclic elastostatic compression (prestrained by viscoplastic or viscoelastic responsive strain) and compared the obtained results with the diffusion of Fe tracer and constituent Zr, Ti and Al atoms in the as-cast BMGs. Table 2 shows chemical composition analysis results for as-cast, deformed and cold-rolled Zr_55_Ti_5_Al_10_Cu_20_Ni_10_ BMGs with tracer Fe layer coating, respectively.

**Table 2:** Chemical composition analysis results for as-cast, deformed and cold-rolled Zr_55_Ti_5_Al_10_Cu_20_Ni_10_ BMGs with tracer Fe layer coating, respectively.

at.%	Zr	Ti	Al	Cu	Ni	Fe
Nominal	55	5	10	20	10	—
As-cast	51.59 ± 0.02	4.50 ± 0.01	10.78 ± 0.01	22.36 ± 0.02	9.93 ± 0.02	0.84 ± 0.02
Deformed	50.14 ± 0.02	4.45 ± 0.01	10.55 ± 0.01	23.36 ± 0.02	10.55 ± 0.02	0.95 ± 0.02
Rolled	51.76 ± 0.02	4.62 ± 0.01	10.82 ± 0.01	21.93 ± 0.02	10.03 ± 0.02	0.84 ± 0.02

**Figure 3:**
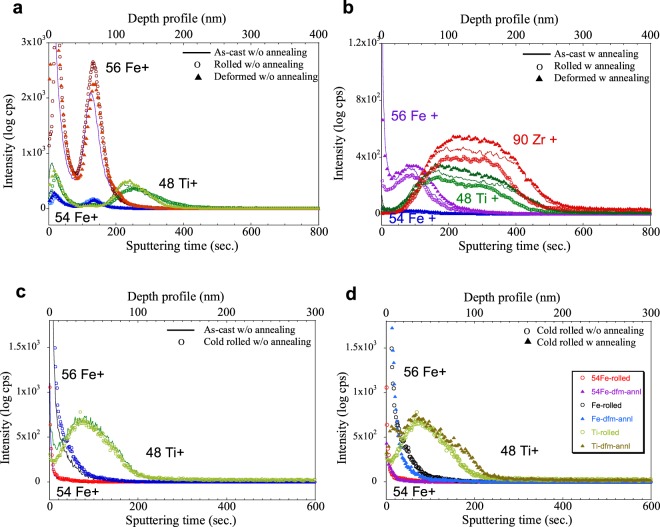
(**a**) SIMS diffusion profiles of external tracer atoms and constituent elements obtained for as-cast, (solid lines), cold-rolled (open circles), and elastostatically compressed (solid triangles) Zr_55_Ti_5_Al_10_Cu_20_Ni_10_ specimens measured in the as-deposited state before diffusion annealing. (**b**) SIMS diffusion profiles of external tracer atoms and constituent elements obtained for as-cast, (solid lines), cold-rolled (open circles), and elastostatically compressed (solid triangles) Zr_55_Ti_5_Al_10_Cu_20_Ni_10_ specimens measured after annealing at 500 K for 120 min. (**c**) SIMS diffusion profiles of external tracer atoms and constituent elements obtained for as-cast, (solid lines) and cold-rolled (open circles) Zr_44_Ti_11_Cu_9.8_Ni_10.2_Be_25_ specimens measured in the as-deposited state before diffusion annealing. (**d**) SIMS diffusion profiles of external tracer atoms and constituent elements obtained for as-cast, (solid lines) and cold-rolled (open circles) Zr_44_Ti_11_Cu_9.8_Ni_10.2_Be_25_ specimens measured in the as-deposited state after annealing at 523 K for 120 min.

The SIMS analyses results in Fig. 3(a) show concentration profiles of the Fe tracer atoms and the constituent element Ti obtained independently from three different samples before diffusion annealing for as-cast (solid lines), cold-rolled (open circles) and elastostatically compressed (solid triangles) Zr_55_Ti_5_Al_10_Cu_20_Ni_10_ (at.%) BMGs, respectively. As expected, there is no significant difference of the concentration profiles of Fe and Ti for the as-deposited state before diffusion annealing. Figure 3(b) shows the concentration profiles of the Fe tracer and the constituent elements Ti, Zr, Al obtained from three different samples after diffusion annealing at 500 K for 2 h, respectively. Each curve corresponds to the constituent elements of a single sample. Penetration profiles for Fe diffusion in the Zr-based BMGs [Zr_55_Ti_5_Al_10_Cu_20_Ni_10_ (at.%)] are shown in Fig. 3(b), where the activity, being proportional to the tracer concentration, is plotted versus the elapsed time (depth) of penetration. Interestingly, the elastostatically deformed BMG line (solid triangles) shows that only the penetration elapse time of the Fe concentration upon diffusion is longer than for the as-cast and cold-rolled materials. The elastostatically compressed BMG (represented by solid triangles) displays an acceleration of diffusion compared to the cold-rolled (represented by open circles) or as-cast BMG samples (represented solid lines). The diffusivity of Fe atoms at the surface is faster in the elastostatically deformed sample than for the rolled sample, and the effect of strain on the difference in the diffusivity between the deformed and the rolled sample is significant at this stage due to the contribution of plastic strain in the rolled sample to the overall governing diffusion process. The diffusion behavior of the constituent elements (Zr, Ti) deduced from the penetration profiles is also shown in Fig. 3(b). The penetration depths of the constituent elements in the elastostatically deformed Zr-based BMG are similar to those of the as-cast glass except the concentration levels during diffusion up to saturation. However, the cold-rolled BMG (represented by open circles) displays a significantly retardation of diffusion compared to the elastostatically compressed (represented by solid triangles) or as-cast BMG (represented by solid lines).

Deformation by viscoplastic or viscoelastic straining is known to be homogeneous rather than being localized as the deformation created upon plastic straining^5,7,27^. Moreover, elastic confinement by the applied strain can form free volume clusters that can enhance diffusion of atoms^10,34^. There are previous reports that the Stokes-Einstein model can be applied to homogeneous deformation of BMGs at high temperature or confined stress conditions^14,36^ and that viscoelastic relaxation is a cooperative mechanism changing the diffusion in metallic glasses^37^. However, even at room temperature, it is also possible to apply the Stokes-Einstein condition to homogeneously deformed BMGs subjected to viscoelastic straining during elastostatic compression. Therefore, the formation of free volume in the BMG by viscoplastic straining will enhance the diffusion of BMG in the elastic regime. On the contrary, plastic strain will retard diffusion in compressively strained regions (surface of rolled samples) but enhances diffusion through fast atomic motion in localized shear bands or tensile strained regions (inside of the rolled sample).

To verify the reproducibility of our results, SIMS analyses were performed [Fig. 3(c,d)], under the same conditions for the as-cast (solid lines) and cold-rolled (open circles) Zr_44_Ti_11_Cu_9.8_Ni_10.2_Be_25_ (at.%) BMGs without any heat treatment, utilizing an as-sputtered Fe layer on the surface of the samples [Fig. 3(c)]. The profiles of the Fe atoms before diffusion annealing are identical for both as-cast and cold-rolled BMGs, even when the samples were cold-rolled to 30% plastic strain. However, as shown in Fig. 3(d), there is a distinct shift of the Fe56 and Fe54 profiles in the range of 20~100 nm for the cold-rolled and annealed samples. The cold-rolled BMG with diffusion annealing (represented by solid triangles) displays a retardation of diffusion compared to the as-cast (represented by solid lines) or cold-rolled BMG without diffusion annealing; as a result, the as-cast BMG line shows penetration depths of the Fe concentration by diffusion that are larger than for the cold-rolled material. The diffusivity of Fe atoms at the surface is faster in the as-cast sample than for the rolled samples, and the effect of strain on the difference in the diffusivity between the as-cast and the rolled sample is contributed from plastic strain in the rolled sample. These results are in good agreement with previously presented observations revealing that compressive residual stresses generated by plastic straining retard the diffusion of Fe tracer atoms near the surface of cold-rolled Zr-based BMG^14^. Furthermore, there is a small “shoulder” of the 48Ti + profile at a distance of 70~210 nm away from the surface for the cold-rolled samples [Fig. 3(d)]. This suggests that the diffusion of Ti constituent atoms is accelerated in the cold-rolled Zr-based BMG due to the internal tensile residual stresses introduced by plastic deformation^14^. These results indicate that the applied mode of strain significantly contributes to the diffusion behavior of Zr-based BMGs. Moreover, there is a different diffusion behavior of constituent atoms and tracer atoms even under the same strain condition. The diffusion coefficients obtained from the SIMS analysis results are summarized in Table 3.

**Table 3:** Diffusion coefficients of tracer atom and constituent elements measured from SIMS analysis results for as-cast, deformed and cold-rolled Zr-based BMGs shown in Fig. 3.

Samples	Status	Zr	Ti	Fe
Zr55	As-cast	2.59 × 10^–20^	2.07 × 10^–20^	2.08 × 10^–21^
	Deformed	2.69 × 10^–20^	2.14 × 10^–20^	3.78 × 10^–21^
	Rolled	2.16 × 10^–20^	1.63 × 10^–20^	2.18 × 10^–21^
Zr44	As-cast	—	4.42 × 10^–21^	4.33 × 10^–22^
	Rolled w/o*	—	4.59 × 10^–21^	5.61 × 10^–22^
	Rolled	—	6.27 × 10^–21^	2.67 × 10^–22^

*as-deposition state without annealing.

**Figure 4:**
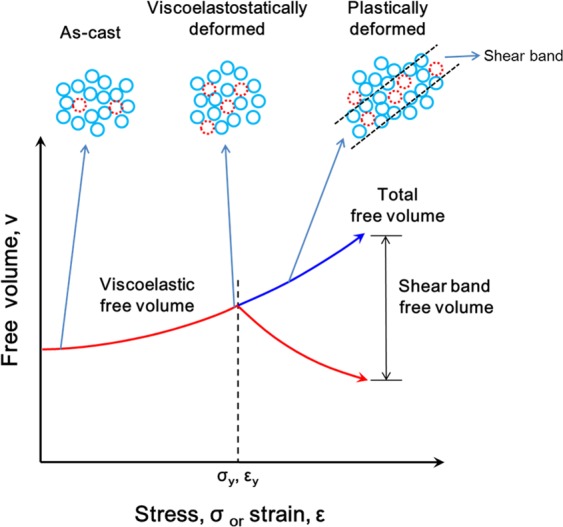
Schematic of the atomistic mechanism in the applied strain and the formation of free volume (loosely packed) regions in bulk metallic glasses under the effect of deformation, i.e., viscoplastic or viscoelastic responsiveally deformed by cyclic elastostatic compression and plastically deformed by multiple cold-rolling. The free volume increases under applied stress by the contribution of viscoplastic or viscoelastic responsive strain which is below the yield-stress and shear dilatation which is above the yield-stress.

As shown in schematic illustration of Fig. 4, the amount of free volume or loosely packed atomic clusters increases upon straining up to the yield stress resulting in change of enthalpy shown in Fig. 1(b). The maximum amount of free volume without localization of strain in the sample (1.1% from DSC measurement) is reached at stresses below the yield stress by elastostatic compression. This is due to the fact that shear band formation causes the formation of free volume localized in shear bands^38^. Therefore, diffusion of tracer Fe atoms in deformed samples is accelerated but retarded in the rolled sample which contains compressive residual stresses. This indicates that the diffusion of the Fe tracer atoms is accelerated by the formation of free volume clusters created by the viscoplastic or viscoelastic responsive strain introduced upon elastostatic compression. The residual stress effect is clearly observed for the diffusion of the constituent atoms in the cold-rolled samples, as shown in Fig. 3(b). However, even though the deformed samples were annealed, the diffusion of the constituent atoms in the deformed samples shows a similar level compared to that of the as-cast material due to formation of a large amount of free volume. Diffusion of the constituent atoms in the severely plastically strained samples subjected to multiple cold rolling steps is also accelerated by the formation of free volume (2.58% from DSC measurement) or tensile residual stress at the internal regions of samples, as shown in Fig. 3(d).

### Atomic size contribution to the diffusivity under different strain modes

During elastostatic compression, we might approximately consider that free volume could be created in the form of a vacant atomic site, i.e. a vacancy-like localized free volume. Ye *et al*. suggested that the formation of free-volume zones surrounded by elastic atomic clusters can dissipate the strain energy during cycling loading^34^. The probability for forming a vacancy for a specific site, i.e. replacing a specific alloying element, is proportional to the atomic fraction of that alloying element under homogeneous flow conditions of the MG^39^. We can consider two different cases of vacancy-mediated diffusion in amorphous alloys: In the first one, the atomic volume (v*) is equal or smaller than the free volume of a vacancy (*v*_*f*_); (v*< v_*f*_), such as diffusion of the Fe tracer or small constituents such as Be in the present MG. The second case refers to situations where the atomic volume is larger than the free volume of the vacancy (v*≥ *v*_*f*_), such as given by the diffusion of the larger constituents Zr, Ti, Ni, Cu and Al in the present MG^1^.

The diffusion coefficient in a cubic system can be described by the following relation^1,39^,

1 $${D}_{A}=\frac{1}{6}\Gamma {\lambda }^{2},$$

where *Г* is the jump frequency and *λ* is the jump length (≈atomic diameter). We take this equation here as a good approximation for a close-packed amorphous system. For vacancy diffusion, the probability of successful jumps of any given atom in 1 sec is given by *p*_*v*_,

2 $$\Gamma =zv{p}_{v}exp\left(-\frac{\Delta {G}_{m}}{kT}\right),$$

where *ΔG*_*m*_ is the activation energy of motion, *k* is Boltzmann’s constant, *z* is the fraction of potential jump sites, *v* is the frequency of atomic vibration (∝concentration, for most metals ~10^13^)^1^ and *p*_*v*_ is the probability that one site is vacant (≈mole fraction of vacancies). If the vacancies are in the equilibrium state ${p}_{v}={p}_{v}^{e}$

3 $${p}_{v}^{e}=exp\left(-\frac{\Delta {G}_{v}}{kT}\right),$$

where *ΔG*_*v*_ is the activation energy for formation or removal of a vacancy.

In the first case, i.e. diffusion of tracer atoms, such as for Fe diffusion in Zr-based MG (v*< *v*_*f*_), the net number of forward jumps per second per potential jump site without distortional strain is described by^1^:

4 $$\Gamma =zvexp\left(-\frac{\Delta {G}_{m}+\Delta {G}_{v}}{kT}\right).$$

The free energy of the atom after the jump is therefore decreased by *ΔG*_*v*_. Here, the activation energy for a diffusive jump into a neighboring vacancy is not the same as the activation energy for creating a vacancy. Spaepen described that the net number of forward jumps per second can be calculated as the difference between the forward flux over an activation barrier (*ΔG*_*m*_ − *ΔG*_*v*_*/2*) and the backward flux over an activation barrier (*ΔG*_*m*_ + *ΔG*_*v*_*/2*)^1^.

5 $$\Delta {G}_{v}=\tau \varOmega $$

6 $$z=exp\left(-\frac{\gamma {v}^{\ast }}{{v}_{f}}\right)$$

7 $$\Gamma =exp\left(-\frac{\gamma {v}^{\ast }}{{v}_{f}}\right)2{\rm{\nu }}\,\sinh \,\frac{\tau \varOmega }{2kT}exp\left(-\frac{\Delta {G}_{m}}{kT}\right)$$

8 $${D}_{A}={D}_{A0}exp\left(-\frac{\Delta {H}_{m}}{kT}\right),$$

where *v*_*f*_ is the average free volume of an atom (≈1.0 ± 1.3 × 10^−31^ m^3^)^9^, v* is the effective size of atomic volume (=1.17 ± 0.1 × 10^−29^ m^3^ for Fe)^9^, *γ* is the shear strain (≈1), *τ* is the shear stress (=500 MPa) and *Ω* is the atomic volume (=1.46 × 10^−29^ m^3^)^1,9,35^. This can be rewritten as:

9 $$\log \,{D}_{A}=\,\log \,{D}_{A0}-\frac{\Delta {H}_{m}}{k}\left(\frac{1}{T}\right).$$

From Eq. (9) the diffusion coefficient without strain *D*_*A*_ can be determined as a function of temperature, i.e., when *log D*_*A*_ is plotted against (*1/T*) a straight line is obtained with a slope equal to (*–ΔH*_*m*_*/k*) and an intercept on the *log D*_*A*_ axis at *log D*_*A0*_. From previous studies^10,14^, we can use some values for our present investigations. The diffusivity *D*_*A0*_ of tracer Fe atoms in as-cast Zr_44_Ti_11_Cu_9.8_Ni_10.2_Be_25_ (at.%; Zr44) BMG is (D_as-cast_ = 5.0 ± 0.75 × 10^−23^ m^2^/s at 525 K)^14,37^, the atomic size (diameter) of the tracer Fe is 0.248 nm, which is the smallest atomic size comparing to the constituent elements Zr (dZr = 0.320 nm), Ti (dTi = 0.294 nm), Cu (dCu = 0.256 nm), Ni (dNi = 0.250 nm), Al (dAl = 0.286 nm) except Be (dBe = 0.226 nm), respectively^10,14^. From Eq. (11), we can also estimate the entropy change of the moving vacancy (*ΔS*_*m*_ = −2.36 × 10^−22^ J/K) with an activation energy (*ΔH*_*m*_ = −6.35 × 10^−22^ J/g) from the slope (*−ΔH*_*m*_*/k* = 46.06 K/g) of *log D*_*A*_ against (*1/T*).

From Eq. (9) it follows that:

10 $${D}_{A0}=\left[\frac{1}{3}{\lambda }^{2}\nu exp\left(-\frac{\gamma {v}^{\ast }}{{v}_{f}}\right)\sinh \,\frac{\tau \varOmega }{2kT}exp\left(\frac{\varDelta {S}_{m}}{k}\right)\right]$$

11 $$\Delta {S}_{m}=k\,\log \left(\frac{3{D}_{A0}}{{\lambda }^{2}\nu \,\sinh \,\tau \varOmega /2kT}\right),$$

where *Ω* = 1.46 × 10^−29^ m^3^, *k* = 1.38 × 10^−23^ J/K, *v* = 10^13^ s^−1^, T = 300 K, *τ* = 500 MPa and *λ* is the average diameter (≈ the jump length) of constituent elements (≈ 2.52 ± 0.65 × 10^−9^ m)^1,9,35^. Therefore, from Eqs. (9) and (10) the diffusivity without atomic distortion strain during diffusion can be calculated for viscoplastic or viscoelastic responsiveally strained Zr-based BMG as *D*_*A0*_ = 3.84 ± 0.22 × 10^−15^ m^2^/s and *D*_*A*_ = 4.64 ± 0.4×10^−7^ m^2^/s at 300 K, respectively.

However, these diffusion coefficients estimated based on the vacancy mechanism are unrealistically high for the diffusion process, because the vacancy model assumes that the activation energy barrier for vacancy formation and vacancy annihilation are equal^1^. In a simple diffusion process, when an atom makes a diffusive jump into a vacancy, the vacancy just changes position with the diffusing atom. Thus, the energy barrier comes just from the saddle point configuration when the diffusing or jumping atom is just between two neighboring atoms, i.e. at the position of maximum elastic distortion during the jump^40,41^. Therefore, this large difference comes from the fact that the activation energy for a diffusive jump into a neighboring vacancy is not the same as the activation energy for creating a vacancy used in this vacancy model.

Therefore, diffusion of small penetrant in amorphous materials is applicable transition state model^40,41^. The strain energy necessary to expand the volume (*Δv*) by shuffling a tracer atom into the original volume of an atom (*v*) can be approximated by the elastic distortion energy. The elastic distortion strain energy difference (*ΔG*_*e*_) between the minimum and the transition-state saddle point by elastic distortion can be calculated by the following relations^1,42^:

12 $$\Delta {G}_{e}=S\frac{\Delta {v}^{2}}{v}$$

13 $$S=\frac{2}{3}\mu \frac{1\,+\,{v}_{p}}{1\,-\,{v}_{p}},$$

where Δ*G*_*e*_ is the elastic distortion energy, *μ* is the shear modulus (=3.29 × 10^10^ N/m^2^) and *ν*_*p*_ is Poisson’s ratio, usually taken as ≈0.4 for metallic glass^1^.

From the transition state theory, if the distance between amorphous matrix atoms is increased by (*r*_*p*_), the space left over is termed accessible volume (*Δv*). The accessible volume for a moving atom of radius (*Δr*) is defined as the volume in the amorphous matrix traced out by the atom’s center for all the regions in which the atom can fit^41^. The radius of the moving atom is given by^41^:

14 $${r}_{p}={2}^{1/6}(\frac{\sigma }{2}),$$

where *σ* is the Lennard-Jones diameter of the particular atom, which can be considered as the initial distance of the matrix atoms^41^. The difference in radius (*Δr*), which is the half of expanded distance of the matrix atoms by the moving atom, can be described as:

15 $$\varDelta r=\frac{1}{2}\sigma ({2}^{1/6}-1),$$

where 1/2*σ* ≈ average radius of the matrix atoms and *Δr* ≈ radius of the Fe tracer atom (dFe/2),

16 $$\varDelta v=\frac{4}{3}\pi {\left(\frac{\sigma }{2}+\Delta r\right)}^{3}\,and\,v=\frac{4}{3}\pi {\left(\frac{\sigma }{2}\right)}^{3}.$$

Then Eq. (12) can be described as following:

17 $$\Delta {G}_{e}=\left(\frac{2}{3}\mu \frac{1\,+\,{v}_{p}}{1\,-\,{v}_{p}}\right)\frac{32{(\sigma /2+\Delta r)}^{6}}{3{\sigma }^{3}},$$

where *ΔG*_*e*_ is the elastic distortion energy, *μ* is the shear modulus (=3.29 × 10^10^ N/m^2^) and *ν*_*p*_ is Poisson’s ratio, usually taken as ≈0.4 for metallic glass^1^.

The transition state model provides an approximation to calculate the movement of the diffusing atom from the initial state cavity through the transition state neck to a neighboring cavity, which causes a rearrangement of the involved clusters^40,41^. The rate coefficient or frequency for the jump (*Г*) in Eq. (2) is given by the transition state equation:

18 $$\Gamma =\frac{kT}{h}\frac{Q{\prime} }{Q}exp\left(-\frac{\Delta {G}_{e}}{kT}\right),$$

where *Q’* is the partition function of the transition state and *Q* is the partition function of the diffusing atom, respectively. If the frequency distributions of the moving atoms and the transition state are identical, then *Q’*/*Q* = 1. The most primitive approximation is then to assume that there is no difference between *Q’* and *Q*, in which case the frequency factor (*kT/h* = *ν*) is approximately given by ~10^13^ s^−1^ in Eq. (2)^1,41^.

The diffusion coefficient expressed by Eq. (1) can be modified in the transition state model in the following form:

19 $${D}_{A}=\frac{1\times {10}^{13}}{6}exp\left(-\frac{\Delta {G}_{e}}{kT}\right){\lambda }^{2}.$$

From Eq. (19) the diffusivity of Fe tracer atoms in viscoplastic or viscoelastic responsiveally strained Zr-based BMG can be estimated as *D*_*A*_ = 1.08 ± 0.4 × 10^−21^ m^2^/s at 300 K. This value is very close to the diffusivity measured from SIMS analysis in Table 3, *D*_*Fe-deform*_ = 3.78 ± 0.01 × 10^−21^ m^2^/s. Hence, the diffusion behavior of Fe tracer atoms in elastostatically deformed Zr-based BMG can be realistically explained by the transition state model rather than by the vacancy model.

In the second case, i.e. diffusion of the constituent atoms, such as Zr diffusion in Zr-based MG (v*≥ *v*_*f*_), the net number of forward jumps per second per potential jump site with distortional strain can be described by^1^:

20 $$\Gamma =zvexp\left(-\frac{\Delta {G}_{m}+\Delta {G}_{v}+\Delta {G}_{e}}{kT}\right).$$

The energy necessary to squeeze an atom with volume v* into a smaller hole of volume *v* (or *v*_*f*_) can be approximated by the elastic distortion energy which can be calculated by eqs, (12) and (13)^1,42^. This gives:

21 $$\Gamma =exp\left(-\frac{\gamma {v}^{\ast }}{{v}_{f}}\right)2{\rm{\nu }}\frac{kT}{S}\left[\cosh \left(\frac{\tau \varOmega }{2kT}\right)-1\right]exp\left(-\frac{\Delta {G}_{m}}{kT}\right)$$

22 $${D}_{A\ast }={D}_{A0\ast }exp\left(-\frac{\Delta {H}_{m}}{kT}\right)$$

23 $$\log \,{D}_{A\ast }=\,\log \,{D}_{A0\ast }-\frac{\Delta {H}_{m}}{k}\left(\frac{1}{T}\right)$$

From Eq. (23) the diffusion coefficient under strain *D*_A*_ can be determined as a function of temperature; when *log D*_A*_ is plotted against (*1/T*) a straight line is obtained with a slope equal to (−*ΔH*_*m*_*/k*) and an intercept on the *log D*_A*_ axis at *log D*_A0*_.

The slope (−*ΔH*_*m*_*/k*), which corresponds to the activation energy to move free volume or a vacancy under strained diffusion, [*ΔH*_*m*_ from Eq. (9)] is equal to that of non-strained diffusion [*ΔH*_*m*_ from Eq. (23)]. This shows that the mechanism of moving a vacancy or free volume upon diffusion in metallic glasses does not change and is independent of the strain mode. However, the applied strain can change the appartent diffusivity (*D*_*A*_ or *D*_A*_) in metallic glass due to the increased amount of free volumes which are maximized under viscoplastic or viscoelastic responsive deformation (shown in Fig. 4). We can calculate *D*_A0*_ by Eq. (22) from the entropy obtained (*ΔS*_*m*_ = −2.39 × 10^−22^ J/K) by Eq. (12):

24 $${D}_{A0\ast }=\left\{\frac{1}{2}{\lambda }^{2}\nu \frac{kT(1-{\nu }_{p})}{\mu (1+{\nu }_{p})}exp\left(-\frac{\gamma {v}^{\ast }}{{v}_{f}}\right)\left[\cosh \left(\frac{\tau \varOmega }{2kT}\right)-1\right]exp\left(\frac{\varDelta {S}_{m}}{k}\right)\right\},$$

where *μ* = 3.29 × 10^10^ N/m^2^ at 300 K, *ν*_*p*_ ≈ 0.4 and *γ* ≈ 1^1,41^. Therefore, from Eqs. (22) and (24) the diffusivity with atomic distortion strain during diffusion can be calculated for the viscoplastic or viscoelastic responsiveally strained Zr-based BMG as *D*_A0*_ = 8.63 ± 0.12 × 10^−47^ m^2^/s and *D*_A*_ = 8.35 ± 0.01 × 10^−21^ m^2^/s at 300 K, respectively. This value is slightly lower than the diffusivity measured from SIMS analysis in Table 3, *D*_*Zr-deform*_ = 2.69 ± 0.02 × 10^−20^ m^2^/s. The diffusion behavior of constituent Zr atom in elastostatically deformed Zr-based BMG can be explained by the vacancy model.

**Figure 5:**
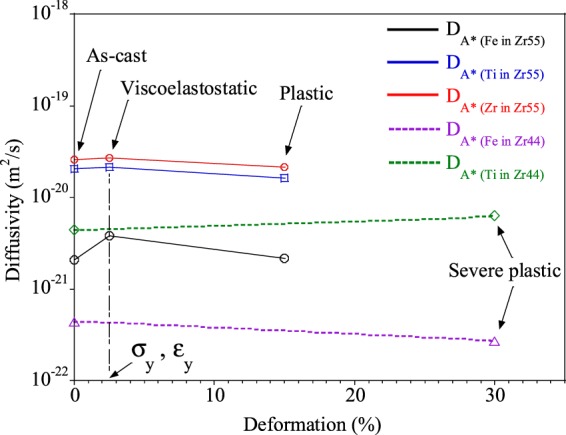
The effect of strain introducing by atomic size on the diffusivity as a function of deformation in metallic glasses at 300 K.

The diffusivity of Fe tracer atoms compared to that of constituent Zr and Ti atoms in different strain states (as-cast, viscoplastic or viscoelastic responsiveally deformed, plastically deformed and severe plastically deformed) is plotted in Fig. 5. The diffusion of the Fe tracer without atomic distortion is expected to show an accelerated motion compared to the as-cast material. These phenomena are confirmed by the results of the SIMS measurements [Fig. 3(b,d)], showing an acceleration of the Fe motion from 2.08 ± 0.22 × 10^−21^ m^2^/s to 3.78 ± 0.01 × 10^−21^ m^2^/s in Zr55 samples subjected to viscoplastic or viscoelastic responsive strain and an retardation of the Fe motion from 4.33 ± 0.22 × 10^−22^ m^2^/s to 2.67 ± 0.01 × 10^−22^ m^2^/s for the Zr44 samples after severe plastic straining, respectively. These changes look relatively small numbers which are probably considered accuracy range of the measurement but we obtained these consistency results from multiple analysis for each samples. The calculated diffusion coefficient of Fe in the Zr-based BMGs without strain differs from the measured value, 5.06 ± 0.75 × 10^−23^ m^2^/s at 525 K^14,37^, due to the difference of the actual strain conditions resulting in atomic structural inhomogeneity or difference of the measuring temperature^14^. It was already shown that the estimated diffusion coefficient value derived from the visco-elastic model is 20% larger than the experimentally measured value^36^. However, the present study can nevertheless give quantitative information based on both experimental and mathematical results about the effect of deformation modes on the diffusion process in Zr-based BMGs. In contrast to the diffusion profile of Fe in Fig. 5, the SIMS profile of the constituent Zr/Ti atoms in the deformed BMG samples shows the same behavior as that of the as-cast BMG, as displayed in Fig. 3(b). This can be explained by the extra strain of atomic distortion created by the larger atomic size of the Zr/Ti atoms, as shown in Fig. 5. Eventually, the diffusion of Zr in plastically deformed samples is significantly slower (2.16 ± 0.4 × 10^−20^ m^2^/s) compared to that of Zr (2.69 ± 0.4 × 10^−20^ m^2^/s) in the viscoelastostatically deformed material. These estimations give indirect evidence that the amount of free volume without localization is maximized by the strain created upon elastostatic compression below the yield stress (cf. Fig. 4). This amount of free volume without shear localization contributes to the enhanced diffusion of tracer atoms. Moreover, the maximum amount of free volume with shear bands contributes the enhancement of diffusion of constituent atoms in severe plastically deformed samples. The atomic size together with the strain mode (viscoelastically, viscoplastically or plastically) play a key role in determining the apparent diffusion in deformed BMG.

In conclusion, the diffusion of tracer atoms, i.e., Fe, in BMG is accelerated by viscoplastic or viscoelastic responsive strain and retarded by inelastic or plastic strain created upon deformation. However, the diffusion of the constituent atoms, i.e., Ti, Zr, in the BMG shows a behavior similar to that of the as-cast sample; diffusion is retarded by plastic strain originating from compressive residual stresses or structural distortion imposed during deformation. The diffusion of tracer atoms, i.e., Fe, in the bulk metallic glass can be mainly described by the transition state model. In contrast, the diffusion of constituent atoms, i.e., Ti, Zr, in the bulk metallic glass is mainly governed by the vacancy model. This reveals that the underlying strain conditions (plastic and viscoplastic or viscoelastic responsive strain created upon multiple cold rolling or cyclic elastostatic compression, respectively) influence the diffusion behavior in different ways. These findings indicate that the local structural changes of BMG that occur during straining depend sensitively on both the applied strain conditions and the alloying elements.

## Methods

Zr_55_Ti_5_Al_10_Cu_20_Ni_10_ (at.%; Zr55) and Zr_44_Ti_11_Cu_9.8_Ni_10.2_Be_25_ (at.%; Zr44) BMGs were selected for the present study because they have been widely characterized and are well-known bulk metallic glasses^15,16,18^. Rectangular bars of 2 × 2 × 4 mm^3^ were cold-rolled multiple times up to 15% (Zr55) and 30% (Zr44) plastic strain, with 0.05 mm reduction in thickness for each pass. Slab-shape bars of 2.06 × 2.52 × 4.57 mm^3^ were elastostatically loaded for 30 cycles up to 85% of the yield strength by uniaxial compression at a strain rate of 1×10^−5^ s^−1^ with 2.5% thickness reduction after cyclic uniaxial compression. The mechanical properties of the as-cast and differently pre-strained specimens were characterized by compression testing using an Instron 8563 electromechanical machine at an initial strain rate of 3×10^−4^ s^−1^. Compression test specimens with an aspect ratio of 2:1 (h:a) were prepared by cutting and polishing the deformed bars according to the ASTM standard (E9). The compressive loading was imposed on the specimens along the rolling direction (RD).

The microstructures and chemical compositions were analyzed by a field emission scanning electron microscope (FE-SEM, Lectropol 5) with electron probe microanalysis (EPMA, SX 100). Structural characterization of the samples was performed using a Philips APD 3520 X-ray diffractometer with monochromatic Cu-Kα radiation. The thermal characterization of the samples was performed by a differential scanning calorimeter (DSC; Perkin-Elmer Pyris-1). The density of the samples was determined by the Archimedes method using a Mettler Toledo-AT261 Delta-Range density measurement system.

The as-cast and rolled samples, two samples of each condition, for the diffusion experiment were prepared by carefully controlled surface condition followed micro-polishing and Ar ions sputtered cleaning under <10^−9^ mbar. A Fe diffusion layer was deposited up to 200 nm thickness with a deposition rate of 0.05 nm/s during 1 h 30 min/sample resulting in 0.84~0.95 at.% onto the surface. Diffusion anneals were performed at 523 K (Zr44) and 500 K (Zr55) for 7200 s (2 h), respectively, which was selected temperatures below the glass transition (*T*_g_)^14,41,43^. The annealing temperature was controlled with an accuracy of ±1.5 K. Details of sample preparation described in elsewhere^14^. Depth profiles of constituent elements and tracer atom concentration were measured by secondary ion mass spectrometry (SIMS; TOF-SIMS5 model of ION-TOF in analysis mode by Bi1+ gun with 25 keV and 1 pA, rater size 100 μm × 100 μm) with 1 mm^2^ scanning area in 3 different regions of each sample.
